# Supplementary material for: Stakeholder perceptions of bird-window collisions
Source: PLoS One. 2022 Feb 10;17(2):e0263447. doi: 10.1371/journal.pone.0263447 (PMC8830717; doi:10.1371/journal.pone.0263447)
Supplement: S1 File — Strengths, weaknesses, opportunities, and threats (SWOT) survey distributed to all respondents (i.e., Survey 1 described in main text) consisting of all pairwise comparisons between factors in each SWOT category using a scale of one to nine. For this survey, all possible pairwise comparisons were made between factors within (but not between) each SWOT category (e.g., all strengths compared to each other, but strengths not compared to weaknesses, opportunities, and threats). Analysis of responses to this survey revealed top-ranked SWOT factors in each category, which were unique to each stakeholder group and used to generate comparisons in Survey 2. (PDF) [file pone.0263447.s001.pdf]

Title: Perceptions and Priorities of Bird-Window Collision Mitigation and Prevention

Principle Investigator: Georgia Riggs

Purpose: The main goal of this study is to gain insight into the perceptions and priorities of major stakeholders regarding bird-window collision mitigation and prevention.

What to Expect: I am requesting your help with my research that aims to create a body of knowledge on human perceptions of bird-window collisions. Please take approximately 10 minutes to complete the questionnaire titled "Perceptions and Priorities of Bird-Window Collision Mitigation and Prevention". Please note that there is no 'right' or 'wrong' answer. I am simply interested in your opinion.

Risks: There is minimal risk associated with this project, which is expected to be no greater than that ordinarily encountered in daily life.

Benefits: There are no direct benefits to you. However, the study results will provide insight into reducing bird-window collisions.

Compensation: There is no financial compensation.

Your Rights and Confidentiality: Your participation in this research is voluntary. There is no penalty for refusal to participate, and you are free to withdraw your consent and participation in this project at any time.

Confidentiality: I will ensure to protect confidentiality of respondents. The aggregate data will be used in any related reports/publications/presentations. I will never report respondent names in any reports. Research records will be stored on a password protected computer in a locked office and only the researcher will have access to the records. Data will be destroyed three years after the study has been completed.

Contacts: You may contact Principle Investigator (Georgia Riggs) at the following address and email, should you desire to discuss your participation in the study and/or request information about the results of the study: Georgia Riggs, Masters student, 008C Ag Hall, Dept. of Natural Resource Ecology and Management, Oklahoma State University, Stillwater, OK 74078, Email: georgia.riggs@okstate.edu. If you have questions about your rights as a research volunteer, you may contact the OSU IRB Office at 223 Scott Hall, Stillwater, OK 74078, +1-405-744- 3377 or irb@okstate.edu.

If you choose to participate: Completing the survey through the online Qualtrics program indicates your willingness to participate in this research study.

Block 1

Section A: In this section, I would like to know about your stakeholder group.

Please describe the stakeholder group you are participating as a member of for this survey. If you do not see yours, please select that which most closely aligns to you.

- ○ Architect
- ○ Government biologist
- ○ Homeowner
- ○ Non-Governmental Organization Employee

Block 2

Section B: In this section, I will introduce the issue and study goals.

Bird-window collisions are a major source of human-caused avian mortality. Birds collide with glass due to their inability to perceive it as a barrier and can be stunned or killed upon impact with glass. When birds are stunned, they are vulnerable to threats such as predation. There are multiple solutions to mitigate (reduce) and prevent bird-window collisions. Some mitigation practices include retrofitting existing windows with exterior nets, shades, films, or markers. Examples of prevention practices include designing bird-friendly buildings with less glass, glass behind a type of screening, or patterned or frosted glass. While legislation is not widespread, multiple cities in the US and Canada have adopted legislation that includes bird-friendly design standards for new buildings. The main goal of this study is to gain insight into the perceptions and priorities of major stakeholders regarding bird-window collision mitigation and prevention. Please utilize the table below to compare different attributes associated with bird-window collision mitigation and prevention.

**Strengths (internal)**	**Weaknesses (internal)**
Fewer collisions	No economic incentives building for bird-friendly buildings
Fewer carcasses to clean up	Lack of architect experience in bird-friendly design
Fewer people witnessing collisions	Lack of availability of expert consultation for bird-friendly design
Fewer stunned birds that die of other causes while recovering from colliding	Financial burden of treating glass or including bird-friendly design in building process
**Opportunities (external)**	**Threats (external)**
Recovering bird populations	Unknown social acceptance of bird-friendly treatments and design
Public exposure to bird-friendly options	Lack of understanding of federal/state policy on bird-window collisions
Consideration of birds in building design becoming a norm/standard	Reduced resources available to spend on other facilities maintenance/improvements
Greater energy efficiency of buildings	No federal/state policy in many areas

Block 3

B1: Strengths

Please carry out a pairwise comparison of the following set of factors that are likely to be considered Strengths of bird-window collision mitigation and prevention. Please mark the factor you think is more important than the other. For example, compare the factor "Fewer collisions" with "Fewer carcasses to have to clean up" and mark the option in the direction that accurately reflects the degree of your opinion. Please note there is no 'right' or 'wrong' answer, we are simply interested in your opinion.

	Extremely Important	Very Important	Moderately Important	Slightly Important	Equally Important	Slightly Important	Moderately Important	Very Important	Extremely Important	
Fewer collisions	○	○	○	○	○	○	○	○	○	Fewer carcasses to clean up
Fewer collisions	○	○	○	○	○	○	○	○	○	Fewer people witnessing collisions
Fewer collisions	○	○	○	○	○	○	○	○	○	Fewer stunned birds that die of other causes while recovering from colliding
Fewer carcasses to clean up	○	○	○	○	○	○	○	○	○	Fewer people witnessing collisions
Fewer carcasses to clean up	○	○	○	○	○	○	○	○	○	Fewer stunned birds that die of other causes while recovering from colliding
Fewer people witnessing collisions	○	○	○	○	○	○	○	○	○	Fewer stunned birds that die of other causes while recovering from colliding

Block 4

B2: Weaknesses

Please carry out a pairwise comparison of the following set of factors that are likely to be considered Weaknesses of bird-window collision mitigation and prevention. Please mark the factor you think is more important than the other. For example, compare the factor "No economic incentives for bird-friendly buildings" with "Lack of architect experience in bird-friendly design" and mark the option in the direction that accurately reflects the degree of your opinion. Please note there is no 'right' or 'wrong' answer, we are simply interested in your opinion.

	Extremely Important	Very Important	Moderately Important	Slightly Important	Equally Important	Slightly Important	Moderately Important	Very Important	Extremely Important	
No economic incentives for bird-friendly buildings	○	○	○	○	○	○	○	○	○	Lack of architect experience in bird-friendly design
No economic incentives for bird-friendly buildings	○	○	○	○	○	○	○	○	○	Lack of availability of expert consultation for bird-friendly design
No economic incentives for bird-friendly buildings	○	○	○	○	○	○	○	○	○	Financial burden of treating glass or including bird-friendly design in building process
Lack of architect experience in bird-friendly design	○	○	○	○	○	○	○	○	○	Lack of availability of expert consultation for bird-friendly design
Lack of architect experience in bird-friendly design	○	○	○	○	○	○	○	○	○	Financial burden of treating glass or including bird-friendly design in building process
Financial burden of treating glass or including bird-friendly design in building process	○	○	○	○	○	○	○	○	○	Lack of availability of expert consultation for bird-friendly design

Block 5

B3: Opportunities

Please carry out a pairwise comparison of the following set of factors that are likely to be considered Opportunities of bird-window collision mitigation and prevention. Please mark the factor you think is more important than the other. For example, compare the factor "Recovering bird populations" with "Public exposure to bird-friendly options" and mark the option in the direction that accurately reflects the degree of your opinion. Please note there is no 'right' or 'wrong' answer, we are simply interested in your opinion.

	Extremely Important	Very Important	Moderately Important	Slightly Important	Equally Important	Slightly Important	Moderately Important	Very Important	Extremely Important	
Recovering bird populations	○	○	○	○	○	○	○	○	○	Public exposure to bird-friendly options
Recovering bird populations	○	○	○	○	○	○	○	○	○	Consideration of birds in building design becoming norm/standard
Recovering bird populations	○	○	○	○	○	○	○	○	○	Greater energy efficiency of buildings
Public exposure to bird-friendly options	○	○	○	○	○	○	○	○	○	Consideration of birds in building design becoming norm/standard
Public exposure to bird-friendly options	○	○	○	○	○	○	○	○	○	Greater energy efficiency of buildings
Greater energy efficiency of buildings	○	○	○	○	○	○	○	○	○	Consideration of birds in building design becoming norm/standard

Block 6

<u>B4: Threats</u>

Please carry out a pairwise comparison of the following set of factors that are likely to be considered Threats of bird-window collision mitigation and prevention. Please mark the factor you think is more important than the other. For example, compare the factor "Unknown social acceptance of bird-friendly treatments and design" with "Lack of understanding of federal/state policy on bird-window collisions" and mark the option in the direction that accurately reflects the degree of your opinion. Please note there is no 'right' or 'wrong' answer, we are simply interested in your opinion.

	Extremely Important	Very Important	Moderately Important	Slightly Important	Equally Important	Slightly Important	Moderately Important	Very Important	Extremely Important	
Unknown social acceptance of bird-friendly treatments and design	○	○	○	○	○	○	○	○	○	Lack of understanding of federal/state policy on bird-window collisions
Unknown social acceptance of bird-friendly treatments and design	○	○	○	○	○	○	○	○	○	Reduced resources available to spend on other facilities maintenance/ improvements
Unknown social acceptance of bird-friendly treatments and design	○	○	○	○	○	○	○	○	○	No federal/state policy in many areas
Lack of understanding of federal/state policy on bird-window collisions	○	○	○	○	○	○	○	○	○	Reduced resources available to spend on other facilities maintenance/ improvements
Lack of understanding of federal/state policy on bird-window collisions	○	○	○	○	○	○	○	○	○	No federal/state policy in many areas
No federal/state policy in many areas	○	○	○	○	○	○	○	○	○	Reduced resources available to spend on other facilities maintenance/ improvements
