# Supplementary material for: Stakeholder perceptions of bird-window collisions
Source: PLoS One. 2022 Feb 10;17(2):e0263447. doi: 10.1371/journal.pone.0263447 (PMC8830717; doi:10.1371/journal.pone.0263447)
Supplement: S2 File — Strengths, weaknesses, opportunities, and threats (SWOT) survey distributed to respondents in the homeowner stakeholder group (i.e., Survey 2 for homeowners described in main text) based on their responses to Survey 1. For this survey, all possible pairwise comparisons were made between the top-ranking factors from each SWOT category for homeowners (e.g., top homeowner strength compared to top weakness, opportunity, and threat). (PDF) [file pone.0263447.s002.pdf]

Title: Survey 2: Homeowner Perceptions and Priorities of Bird-Window Collision Mitigation and Prevention

Principle Investigator: Georgia Riggs

Purpose: The main goal of this study is to gain insight into the perceptions and priorities of major stakeholders regarding bird-window collision mitigation and prevention. Based on stakeholder responses to the first survey, I have designed a second survey, which will help me obtain a quantifiable matrix on strengths, weaknesses, opportunities, and threats associated with bird-window collision mitigation and prevention. **This is the second and final survey in this study.**

What to Expect: I am requesting your help with my research that aims to create a body of knowledge on human perceptions of bird-window collisions. Please take approximately 10 minutes to complete the questionnaire titled "Survey 2: Homeowner Perceptions and Priorities of Bird-Window Collision Mitigation and Prevention". Please note that there is no 'right' or 'wrong' answer. I am simply interested in your opinion.

Risks: There is minimal risk associated with this project, which is expected to be no greater than that ordinarily encountered in daily life.

Benefits: There are no direct benefits to you. However, the study results will provide insight into reducing bird-window collisions.

Compensation: There is no financial compensation.

Your Rights and Confidentiality: Your participation in this research is voluntary. There is no penalty for refusal to participate, and you are free to withdraw your consent and participation in this project at any time.

Confidentiality: I will ensure to protect the confidentiality of respondents. The aggregate data will be used in any related reports/publications/presentations. I will never report respondent names. Research records will be stored on a password protected computer in a locked office and only the researcher will have access to the records. Data will be destroyed three years after the study has been completed.

Contacts: You may contact Principle Investigator (Georgia Riggs) at the following address and email, should you desire to discuss your participation in the study and/or request information about the results of the study: Georgia Riggs, Masters student, 008C Ag Hall, Dept. of Natural Resource Ecology and Management, Oklahoma State University, Stillwater, OK 74078, Email: georgia.riggs@okstate.edu. If you have questions about your rights as a research volunteer, you may contact the OSU IRB Office at 223 Scott Hall, Stillwater, OK 74078, +1-405-744-3377 or irb@okstate.edu.

If you choose to participate: Completing the survey through the online Qualtrics program indicates your willingness to participate in this research study.

Block 1

Section A: Introduction and instructions

This is the second and last survey in this study. Please proceed only if you participated in the first survey.

Recently, you completed a survey as a member of the **Homeowner** stakeholder group that investigated perceptions and priorities of bird-window collision mitigation prevention. Based on the survey responses in the **Homeowner** stakeholder group, the highest ranked strength, weakness, opportunity, and threat are:

**Strength:**	Fewer bird-window collisions
**Weakness:**	Lack of availability of expert consultation for bird-friendly design
**Opportunity:**	Recovering bird populations
**Threat:**	No federal/state policy in many areas

Block 2

Section B: Pairwise Comparison

Please carry out the following pairwise comparisons of the top-ranked factors of bird-window collision mitigation and prevention. These factors were the top choices from respondents in the first survey.

Please mark the factor you think is more important than the other. For example, compare the factor "Fewer collisions" with "No federal/state policy in many areas" and mark the option in the direction that accurately reflects the degree of your opinion. Please note there is no 'right' or 'wrong' answer, we are simply interested in your opinion.

	Extremely Important	Very Important	Moderately Important	Slightly Important	Equally Important	Slightly Important	Moderately Important	Very Important	Extremely Important	
Fewer collisions	○	○	○	○	○	○	○	○	○	Lack of availability of expert consultation for bird-friendly design
Fewer collisions	○	○	○	○	○	○	○	○	○	Recovering bird populations
Fewer collisions	○	○	○	○	○	○	○	○	○	No federal/state policy in many areas
Lack of availability of expert consultation for bird-friendly design	○	○	○	○	○	○	○	○	○	Recovering bird populations
Lack of availability of expert consultation for bird-friendly design	○	○	○	○	○	○	○	○	○	No federal/state policy in many areas
Recovering bird populations	○	○	○	○	○	○	○	○	○	No federal/state policy in many areas
