# Supplementary material for: Stakeholder perceptions of bird-window collisions
Source: PLoS One. 2022 Feb 10;17(2):e0263447. doi: 10.1371/journal.pone.0263447 (PMC8830717; doi:10.1371/journal.pone.0263447)
Supplement: Attachment — Submitted filename: Response to Reviewers.docx [file pone.0263447.s006.docx]

To the editorial staff of *PLOS ONE*:

We again thank the Associate Editor and reviewers for the constructive comments that have assisted us in crafting a second revision of manuscript PONE-D-21-17993, *Stakeholder perceptions of bird-window collisions*. We have addressed the relatively minor additional comments by the Associate editor and Reviewer 1, and we believe that the new version of the manuscript is strengthened. Major changes are summarized immediately below, and a full point-by-point response to all comments is also attached.

Some of the more major revisions include:

- We further clarified the definitions of strengths, weaknesses, opportunities, and threats, including providing specific examples related to bird-window collisions that better illustrate the difference between direct and indirect outcomes (strengths and opportunities, respectively) and obstacles (weaknesses and threats, respectively).
- We revised the future research directions section of the Discussion to highlight the need for additional research on social and behavioral barriers to implementation of bird-window collision management activities.
- We added additional text in the Discussion to acknowledge key study limitations highlighted by Reviewer 1, including related to our convenience-based sampling approach and to the limitation of not collecting demographic information and geographic locations from survey respondents.
- We reviewed the reference list to ensure that it is complete and correct. We edited one existing citation, added two new citations, and shifted two existing citations within the manuscript which caused the numbers of most citations to shift. All reference numbers have been updated.

Again, we appreciate the positive remarks and constructive comments that have contributed to an improved paper. We hope you agree the result is worthy of publication in *PLOS ONE*, and we look forward to further correspondence with you regarding the paper.

Sincerely,

Georgia Riggs, Omkar Joshi, and Scott Loss

Additional Editor Comments (if provided):

Associate Editor:
Both reviewers found the revised manuscript to have addressed their comments. However, one reviewer has several remaining points that need to be addressed. In addition to these points a few minor items are as follows:

Abstract. Please include the main goal and objectives of the research in the Abstract.
**RESPONSE**: **We have revised accordingly to add the specific objectives of the research to the abstract (now found in Lines 29-32). We also revised the abstract to better present specific results and implications/conclusions that align with each of these 3 objectives (Lines 38-47).**

Table 2. Indicate what bold terms mean. Also, table legend should be written as stand alone and explain more of what is in the table.

**RESPONSE**: **We have revised the table legend (now in Lines 350-357) to describe the bold terms and to better elaborate on what the different values mean.**

Acknowledgements. Suggest thanking the anonymous reviewers.
**RESPONSE**: **Thank you for the suggestion. We have added thanks to the handling editor and anonymous reviewers in Lines 648-652.**

Reviewers' comments:
Reviewer #1:

 I’d like to thank the authors for their thorough and thoughtful revisions to the manuscript. I think the manuscript is just about ready for publication, but I do have some more minor suggestions below.
**RESPONSE**: **Thank you again for the positive and constructive comments that have helped us improve the manuscript.**

Abstract
Not sure I understand the distinction between direct and indirect outcomes here- see comment in methods about this but I also think it needs to be clarified better here in the abstract: “respondents made pairwise comparisons between various strengths and weaknesses (direct outcomes related to collision management) and opportunities and threats (indirect outcomes)”

**RESPONSE: Please see also our response to the later comment about this same issue in the Methods. Here in the abstract (Lines 34-38), we revised to add brief definitions and examples that should better clarify the definitions of strengths, weaknesses, opportunities, and threats.**

Introduction
Line 100: I think there should be a list of citations here when discussing how SWOT and AHP are commonly used in human dimensions research

**RESPONSE**: **We have added citations to relevant SWOT and AHP research here, and we restructured this sentence to make it clear that the citations support the approach of merging the two analyses. See Lines 108-109.**

Methods
Line 122-124: I see the authors added this sentence to try to clarify the different components of SWOT, but I’m still confused: “Strengths and weaknesses capture direct positive and negative outcomes, respectively, and therefore are considered internal to the issue. Opportunities and threats respectively capture positive and negative indirect outcomes.” What is a “direct outcome” and why are direct outcomes “internal”? What is an “indirect” outcome? I think more detailed explanation of these terms with examples would be helpful.

**RESPONSE**: **We understand that these terms might still cause some confusion, so we have added additional clarifying language, along with examples related specifically to the issue of bird-window collisions, to explain the differences more clearly among strengths, weaknesses, opportunities, and threats (see Lines 128-137). We hope that these additional changes prove clear enough for a broad readership.**

Line 151-152: awkward phrasing here: “they typically have public involvement…”

**RESPONSE**: **We have clarified the wording here to read as (now at Lines 162-165): “they typically engage members of the public through activities such as…”**

Line 163: What emails and social media platforms did you use to reach what types of stakeholders? More detail about this would be helpful so that the reader can better understand the types of biases in this sample (e.g., did you work with certain NGOs or other groups to post on their social media?). I also think its worth mentioning that this second group of people recruited through social media and email (as well as the gateway contacts) is a convenience sample- so basically, you used a combination of snowball sampling and convenience sampling. I also think a sentence or two here on why the convenience and snowball sampling approach (which is usually not sufficient for human dimensions research) is OK in the context of this study/your research questions.

**RESPONSE: Regarding the comment about our use of email and social media platforms, we revised (Lines 174-187) to clarify who we contacted through email and how we did so, and about our approach of sharing recruitment materials (including a recruitment video on YouTube) through Facebook and Twitter.**

**Regarding the comment about convenience sampling: although snowball sampling has been periodically used as a purposive sampling tool to gauge stakeholder perceptions in the conservation social science literature (e.g. Karanth et al. 2008, Said et al. 2016, Krupa et al, 2018), the reviewer is correct that random sampling frameworks have historically dominated mainstream human dimensions research. Concerning our study, it is important to note that the research goal was to depict how similar (or different) the key stakeholder groups were regarding their opinions on bird-window collisions. During the research design stage, we framed our study following the typical protocols of the SWOT-AHP platform, which often uses purposive data collection tools such as in-person focus groups (e.g. Dwivedi and Alavalapati 2009) or mixed-mode sampling with in-person meetings for some stakeholders and web surveys for others (e.g. Starr et al. 2019). However, since we did not have a comprehensive list of stakeholders, we utilized snowball sampling, gateway contacts, and targeting of select HOAs in Stillwater to recruit survey participants. Nonetheless, as already described in Lines 332-337 of the revised manuscript, the range of consistency ratios (CR) for our response data is sufficient to discern the internal consistency of stakeholder opinions. Our study results should therefore broadly represent consensus opinions of each stakeholder group. To address this reviewer comment, we revised the limitations section of the Discussion (Lines 566-568) to acknowledge that our convenience-based sampling contributed to the issue of representativeness of respondents (which we had already discussed in relation to limited sample size). Later in the same paragraph we also had described how consistency ratios indicate that our results should be reliable despite these issues related to convenience sampling and representativeness.**

**References:**

**Said, A., Tzanopoulos, J., & MacMillan, D. (2016). Bluefin tuna fishery policy in Malta: the plight of artisanal fishermen caught in the capitalist net. Marine Policy, 73, 27-34.**

**Karanth, K. K., Kramer, R. A., Qian, S. S., & Christensen Jr, N. L. (2008). Examining conservation attitudes, perspectives, and challenges in India. Biological Conservation, 141(9), 2357-2367.**

**Krupa, M., Cenek, M., Powell, J., & Trammell, E. J. (2018). Mapping the stakeholders: Using social network analysis to increase the legitimacy and transparency of participatory scenario planning. Society & Natural Resources, 31(1), 136-141.**

**Dwivedi, P., & Alavalapati, J. R. (2009). Stakeholders’ perceptions on forest biomass-based bioenergy development in the southern US. Energy Policy, 37(5), 1999-2007.**

**Starr, M., Joshi, O., Will, R. E., & Zou, C. B. (2019). Perceptions regarding active management of the Cross-timbers forest resources of Oklahoma, Texas, and Kansas: A SWOT-ANP analysis. Land Use Policy, 81, 523-530.**

Lines 185-186: Did you create the original list of SWOT factors based on a review of the literature? Talking with stakeholders? Some citations and explanation would be helpful.
**RESPONSE: We revised the manuscript to clarify (now in Lines 203-206) that we developed this initial list of candidate SWOT factors based on our extensive subject matter expertise, which includes familiarity with the scientific and gray literature on this topic, and our years of interactions and collaborations with key stakeholders in federal and state agencies and non-profit conservation organizations. As already stated in the manuscript, we also sought feedback from 3 external experts to create the final list of SWOT factors used in this study.**

Line 238: What is meant by “periodic reminders”? There are standard practices for when/how often to send reminders for surveys to increase response rate (see Dillman)- to what extent were these followed?
**RESPONSE: We revised to clarify (now in Lines 258-260) that, for all stakeholder groups and sampling approaches, we sent one reminder to complete the survey two weeks after the original participation request (we also added a citation to the Dillman reference supporting this follow-up method).**

Line 307-311: I think the lack of data on who the respondents are (particularly with respect to geography) is a major limitation of this research. Collecting personally identifiable information is often done in HD research and is standard practice. I realized this can’t be changed at this point, but was there any information about respondents you could share here to give the reader a sense of who your sample is? Maybe at least providing demographic information about the gateway respondents? I know sample representativeness is brought up a bit in the discussion, but I think this limitation could be more strongly addressed here and in the discussion.

**RESPONSE: Thanks for the opportunity to respond to and clarify this issue. Following well-documented AHP protocols, several researchers have used a very small number of expert opinions to identify stakeholder perceptions regarding natural resource issues. For example, Shrestha et al. (2004) used opinions of three leaders, a research/extension specialist (to represent agriculture research/extension professionals) and two ranchers (to represent landowners), to explore the potential for silvopasture adoption in south-central Florida (page 190, Shrestha et al. 2004). Similarly, Kurtitila et al. (2000) used opinions of two experts to compare strengths, weaknesses, opportunities, and threats associated with forest certification in eastern Finland (page 47, Kurtitila et al. 2000). Masozera et al. (2006) used a total of 11 participants representing three groups (5 from one group and 3 from the other two groups) in their effort to assess suitability of community-based management for the Nyungwe Forest Reserve in Rwanda. In other words, a small sample size is acceptable as long as it meets the consistency ratio (CR) criterion. In these typical studies, researchers do not report personally identifiable information as the sample size is extremely low. Although the sample size for our study (n=52 for the first survey and n=33 for the second survey) is larger than the above-highlighted studies, we unfortunately did not have a pre-established contact list for our respondents and we did not know how many respondents would actually fill out the survey. Therefore, we did not ask for any personally identifiable information. In retrospect, our readers would benefit from this information, and we recognize this lack of information as a limitation. To address this comment, we have added acknowledgment of this limitation in the Discussion (Lines 594-598). Specifically, we note that we did not collect demographic information from respondents, nor did we know the geographic representation of our sample other than for gateway contacts. We then highlight that future research could evaluate how perceptions about bird-window collisions vary regionally and in relation to various demographic factors, since the factors that influence behaviors, perceptions, and conflicts can vary regionally and among demographic groups (e.g., among different age groups).**

**References:**

**Kurttila, M., Pesonen, M., Kangas, J., & Kajanus, M. (2000). Utilizing the analytic hierarchy process (AHP) in SWOT analysis—a hybrid method and its application to a forest-certification case. Forest policy and economics, 1(1), 41-52.**

**Masozera, M. K., Alavalapati, J. R., Jacobson, S. K., & Shrestha, R. K. (2006). Assessing the suitability of community-based management for the Nyungwe Forest Reserve, Rwanda. Forest Policy and Economics, 8(2), 206-216.**

**Shrestha, R. K., Alavalapati, J. R., & Kalmbacher, R. S. (2004). Exploring the potential for silvopasture adoption in south-central Florida: an application of SWOT–AHP method. Agricultural Systems, 81(3), 185-199.**

Discussion

Line 428-430: I think this finding can be more directly stated in the results; I had some trouble finding this exact result in the previous section.
**RESPONSE: Thank you for this suggestion. Although the values supporting this finding were already presented in Lines 339-341 of the revised manuscript, we agree that the comparison could be made more directly, so we added an additional line directly comparing the group priority values for threats between homeowners and conservation practitioners (Lines 345-346).**

Line 470: You mention this study by Menacho-Odio that also is a human dimensions study on bird-window collisions. This makes me think that the statement in the introduction that only one prior study on this topic has been done needs to be revised.
**RESPONSE: Thank you for catching this. We revised this paragraph to note that there have only been two studies evaluating human dimensions of the bird-window collision issue, and we added an additional sentence to highlight the Menachio-Odio study (Line 91-93).**

Line 512: I think it should be mentioned in this paragraph (or elsewhere in the discussion) the need for future research on the additional barriers and motivations to behavior change to design these programs. Some barriers/motivations were examined in this study (i.e. mostly beliefs), but many common factors influencing behavior change (e.g., social norms, behavioral control/efficacy, etc) were not. See the community based social marketing work by McKenzie-Mohr and Kollmuss and Agyeman 2002, Mind the gap: why do people act environmentally and what are the barriers to pro-environmental behavior?

**RESPONSE: We followed the reviewer’s suggestion and revised to add a mention of the need for this type of future research (along with citation to the Kollmuss and Agyeman reference). However, instead of adding this text in the location referenced by this comment, we added it to the later Discussion paragraph that describes several important research needs related to the human dimensions of bird-window collisions (see Lines 584-593).**

Reviewer #2: (No Response)
